# Differences in the Effects of EGCG on Chromosomal Stability and Cell Growth between Normal and Colon Cancer Cells

**DOI:** 10.3390/molecules23040788

**Published:** 2018-03-29

**Authors:** Juan Ni, Xihan Guo, Han Wang, Tao Zhou, Xu Wang

**Affiliations:** School of Life Sciences, The Engineering Research Center of Sustainable, Development and Utilization of Biomass Energy, Yunnan Normal University, Kunming 650500, China; gt_gg30@163.com (J.N.); guoxh1987@163.com (X.G.); wanghan9215@163.com (H.W.); kmzl@163.com (T.Z.)

**Keywords:** epigallocatechin-3-gallate (EGCG), chromosomal instability (CIN), apoptosis, human colon cell

## Abstract

The tea catechin epigallocatechin-3-gallate (EGCG) proved to be the most potent physiologically active tea compound in vitro. It possesses antioxidant as well as pro-oxidant properties. EGCG has the effect of inducing apoptosis of tumor cells and inhibiting cell proliferation. Whether this effect is associated with the antioxidant or pro-oxidative effects of EGCG affecting the genome stability of normal and cancer cells has not been confirmed. Here, we selected Human normal colon epithelial cells NCM460 and colon adenocarcinoma cells COLO205 to investigate the effects of EGCG (0–40 μg/mL) on the genome stability and cell growth status. Chromosomal instability (CIN), nuclear division index (NDI), and apoptosis was measured by cytokinesis-block micronucleus assay (CBMN), and the expression of core genes in mismatch repair (*hMLMLH1* and *hMSH2*) was examined by RT-qPCR. We found that EGCG significantly reduced CIN and apoptosis rate of NCM460 at all concentrations (5–40 μg/mL) and treatment time, EGCG at 5 μg/mL promoted cell division; EGCG could significantly induce chromosome instability in COLO205 cells and trigger apoptosis and inhibition of cell division. These results suggest that EGCG exhibits different genetic and cytological effects in normal and colon cancer cells.

## 1. Introduction

Tea contains high amounts of polyphenols with significant biological activities. Higher tea consumption is associated with beneficial effects in a variety of human diseases [1]. The underlying cellular and molecular mechanisms for the beneficial effects of tea polyphenols have been extensively studied in recent years. The tea catechin epigallocatechin-3-gallate (EGCG) proved to be the most potent physiologically active tea compound in vitro [2]. Structurally, EGCG has hydroxyl groups at carbons 3’, 4’, and 5’ of the B ring and a gallate moiety esterified at carbon 3 of the C ring, which enables EGCG to exhibit predominant antioxidant properties [3].

EGCG has been traditionally regarded as beneficial because of its antioxidant effects, which may aid in a number of clinical conditions such as cancer, obesity, atherosclerosis, diabetes, and neurodegeneration [4]. These age-related diseases are collectively characterized by increased production of reactive oxygen species (ROS) and/or insufficient cellular antioxidant capacity [5,6,7,8,9]. The antioxidant activity of EGCG does not only involve direct trapping of ROS [10,11,12] but also inhibition of ROS production via interaction with anti- and prooxidant proteins [13,14] and chelation of potentially prooxidant metal ions [15,16]. 

Nevertheless, the chemical structure of EGCG makes it susceptible to degradation via auto-oxidation [3]. Auto-oxidation of EGCG generates ROS, with EGCG simultaneously transformed into numerous EGCG auto-oxidation products. The study found that auto-oxidation of EGCG also occurs under cell culture conditions. The half-life of EGCG was less than 30 min in McCoy’s 5A culture media [17]. And Li observed oxidative cellular damages in human lung cancer xenograft tumors after EGCG treatment [18]. This demonstration of prooxidative activities of EGCG in vivo together with the significant role of EGCG-generated H_2_O_2_ in cellular protection against oxidative stress in vitro suggest autooxidation may be as an important component of EGCG-mediated activities [19].

Chromosomal instability (CIN) is defined as an increase in the rate at which whole chromosomes or large chromosomal fragments are gained or lost. It is an important form of genomic instability (GIN). Low levels of CIN can induce cell cycle arrest and allow cells to repair damaged chromosomes. When cells have a serious and irreversible CIN, the cells show a disadvantage in their survival and are eventually cleared by various cell death pathways, such as apoptosis, necrosis, and autophagy [20]. The death of a large number of cells will destroy the body’s tissue homeostasis, leading to a series of diseases; long-term CIN may induce tissue cancer [21]. CIN has long been known to be the underlying cause of human aging [22] and many aging-related diseases such as cancer [23] and Alzheimer’s disease [24], with ROS suggested as causal relationship.

A number of studies in vitro have found that EGCG has the effect of inducing apoptosis of tumor cells and inhibiting cell proliferation [25]. Whether this effect is associated with the antioxidant or pro-oxidative effects of EGCG has not been confirmed. And whether the anti-/pro-oxidative activity of EGCG affects the genome stability of normal and cancer cells differently. In this study, we selected Human normal colon epithelial cells NCM460 and colon adenocarcinoma cells COLO205 to investigate the effects of EGCG on the genome stability and cell growth status of two cell lines and their differences.

## 2. Results and Discussion

### 2.1. The Cytotoxicity of EGCG in COlO205

**Figure 1:**
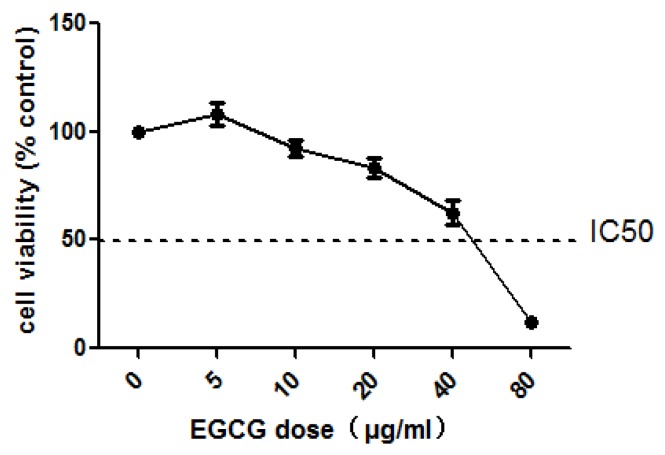
Viability of COLO205 cells in media containing 0, 5, 10, 20, 40, 80 μg/mL EGCG 24 h.

COLO205 cells were exposed to EGCG (5, 10, 20, 40, and 80 μg/mL) for 24 h. The results showed that cell number dropped to 37.33% of control when treated with 40 μg/mL EGCG (Figure 1). In mammalian cell-based genotoxicity tests, the cytotoxicity values of the maximum concentration are allowed to approach but not exceed a reduction of 55%. So, we chose the four concentrations (5, 10, 20, and 40 μg/mL) for follow-up experiments.

### 2.2. The Different Effects of EGCG on the CIN of NCM460 and COlO205 

**Figure 2:**
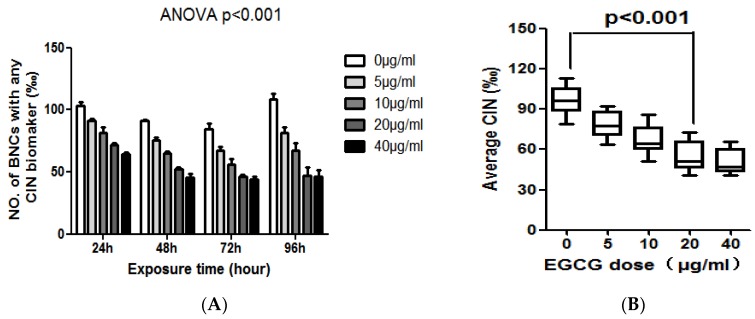
(**A**) The effect of EGCG on the frequency of BNC containing chromosomal instability (CIN) biomarkers (MN and/or NPB and/or NBUD); (**B**) The average effect of 96-h exposure to EGCG at doses of 0, 5, 10, 20, 40 μg/mL on CIN frequency in the NCM460 cell line.

The levels of MN, NPB, and NBUD in NCM460 cells exposed to different concentrations of EGCG for 24, 48, 72, and 96 h are summarized in Table 1. As an overview, EGCG treatment can reduce the CIN of NCM460 cells (*p* < 0.01, Table 1 and Figure 2A). EGCG reduced the frequency of MN, NPB, and NBUD in NCM460 cells by a time-and dose-dependent manner. EGCG had a significant effect on the frequency of MN in NCM460 cells. EGCG significantly decreased the frequency of MN at all concentration and treatment time points in NCM460 cells (*p* < 0.01). The frequency of MN decreased by 61.8% after treatment with 40 μg/mL EGCG for 96 h. We also found there was no significant difference between the 40 μg/mL and 20 μg/mL groups after 96 h treatment (Figure 2B). This result suggested that the declining ability of the highest concentration EGCG (40 μg/mL) on the frequency of MN was decreasing with time.

**Figure 3:**
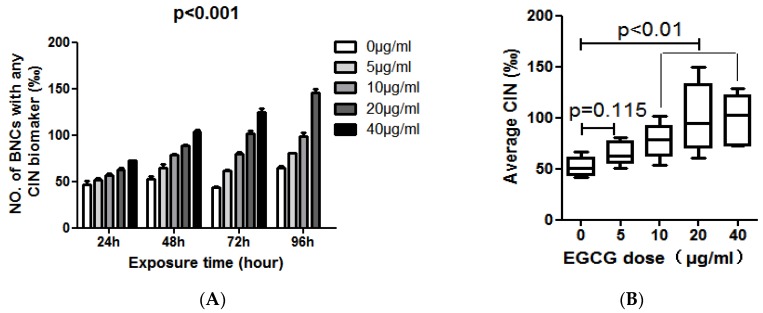
(**A**) The effect of EGCG dose on the frequency of BNC containing chromosomal instability (CIN) biomarkers (MN and /or NPB and/or NBUD); (**B**) The average effect of 96-h exposure to EGCG at doses of 0, 5, 10, 20, 40 μg/mL on CIN frequency in the COLO205 cell line.

At the same time, we observed a different phenomenon in COLO205 cells. The levels of MN, NPB and NBUD in COLO205 cells exposed to different concentrations of EGCG for 24, 48, 72, 96 h were summarized in Table 2. In COLO205 cells, EGCG treatment can significantly increase the CIN including MN, NPB, and NBUD index by a time-and dose-dependent manner (*p* < 0.01, Table 2 and Figure 3A). COLO205 cells were treated with EGCG at 40 μg/mL for 96 h, cell growth was inhibited, NDI = 1.089, not enough binuclear cells to analyze CIN. Considering the effect of EGCG dose on the CIN of COLO205 cells, we found that 10–40 μg/mL of EGCG can significantly increase the CIN of COLO205 cells (*p* < 0.01, Figure 3B).

### 2.3. EGCG Selectively Inhibits the Proliferation and Induces Apoptosis of COLO205 Cell Line

**Figure 4:**
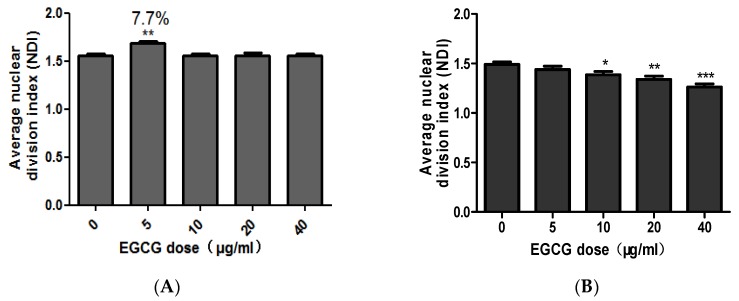
The combined average of the effect of EGCG on NDI in NCM460 (**A**) and COLO205 (**B**). Note: Significant differences between EGCG-treated groups and controls at each treatment interval are indicated by * *p* < 0.05, ** *p* < 0.01 and *** *p* < 0.001.

CBMN-Cyt results showed that low concentration of EGCG (5 μg/mL) could promote the proliferation of NCM460 cells. At four time points of treatment (24, 48, 72, and 96 h), the NDI with EGCG concentration of 5 μg/mL were significantly higher than other treatment groups (*p* < 0.05). Aside from the time, the effect of EGCG on average NDI at each concentration was discussed. Similarly, EGCG at 5 μg/mL significantly promoted the proliferation of NCM460 cells (*p* < 0.01) and the promotion rate was 7.7% (Figure 4A).

In comparison, EGCG can inhibit the proliferation of COLO205 cells. Under the influence of EGCG on average NDI, EGCG at 10, 20, and 40 μg/mL significantly inhibited COLO205 cell division and reduced NDI (*p* < 0.05, Figure 4B).

**Figure 5:**
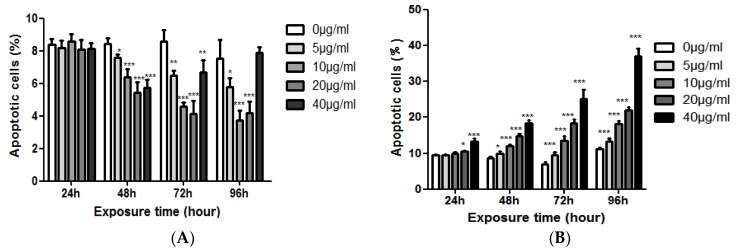
The effects of EGCG dose on apoptotic cells (%) of NCM460 (**A**) and COLO205 (**B**) cells. Significant differences between EGCG-treated groups and controls at each treatment interval are indicated by * *p* < 0.05, ** *p* < 0.01, and *** *p* < 0.001.

The results showed that EGCG had no significant effect on the apoptosis of NCM460 cells when treated with EGCG for 24 h. The apoptotic rate of NCM460 cells was significantly decreased after 48–96 h treatment, and this effect was more obvious with the increase of EGCG concentration, but after 72 h, the inhibitory effect of the highest concentration (40 μg/mL) on the apoptosis of NCM460 cells began to diminish. There was no significant difference between the 40 μg/mL group and the control group after 96 h. But, EGCG could promote the apoptosis of COLO205 cells. With the increase of EGCG concentration and the prolongation of action time, the apoptosis rate of COLO205 cells increased. At 48 h, all the EGCG treatment groups could significantly increase the apoptosis rate (*p* < 0.05, Figure 5).

### 2.4. The Effect of EGCG on the Expression of hMLH1 and hMSH2 in the NCM460 and COLO205 Cell Lines

**Figure 6:**
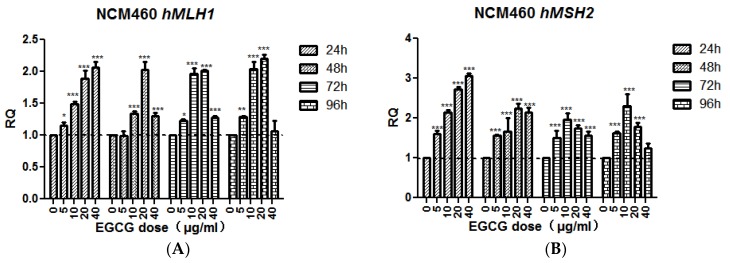
The effect of EGCG on the expression of *hMLH1* (**A**) and *hMSH2* (**B**) in the NCM460 cell line. Bars sharing different letters are significantly different. Significant differences between EGCG-treated groups and controls at each treatment interval are indicated by * *p* < 0.05, ** *p* < 0.01, and *** *p* < 0.001.

The results of qRT-PCR showed that after treated with EGCG for 24 h, the transcription of *hMLH1* and *hMSH2* was gradually increased with the increase of EGCG concentration (*p* < 0.05). After 48 h treatment, the transcriptional level of *hMLH1* gradually increased except the 40 μg/mL, 20 μg/mL reached the highest level (*p* < 0.001), and decreased significantly in 40 μg/mL group (*p* < 0.05). The transcriptional expression of *hMSH2* gradually increased at 5, 10, and 20 μg/mL (*p* < 0.001), and began to decline in the 40 μg/mL group. After 72 h and 96 h, the transcriptional changes of *hMSH2* slightly changed, reaching the highest level (*p* < 0.001) at 10 μg/mL, whereas the 20 μg/mL group began to decline; *hMSH2* transcription levels in 20 and 40 μg/mL groups were significantly down-regulated 96 h after treatment compared with 10 μg/mL groups (*p* < 0.05, Figure 6).

The transcription level of *hMLH1* in EGCG-treated COLO205 cells for 24 h was not significantly different from the control except at 20 μg/mL (*p* < 0.001). After 48, 72, and 96 h treatment, the change tendency was basically the same, first increased then decreased, then increased significantly at 5, 10, 20 μg/mL (*p* < 0.001), reached the highest value at 20 μg/mL, and decreased at 40 μg/mL. The transcription level of *hMLH1* was significantly down-regulated after 40 μg/mL EGCG treatment for 96 h (*p* < 0.001).

**Figure 7:**
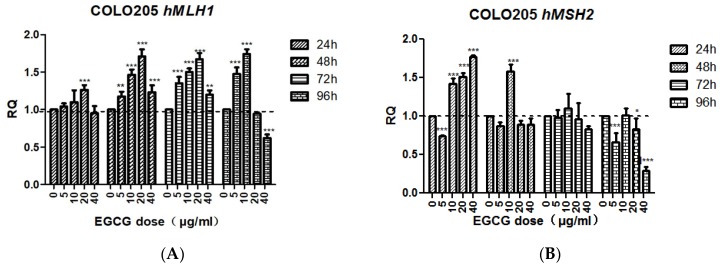
The effect of EGCG on the expression of *hMLH1* (**A**) and *hMSH2* (**B**)*.* Bar sharing different letters were significant differences from each other. Significant differences between EGCG-treated groups and controls at each treatment interval are indicated by * *p* < 0.05, ** *p* < 0.01, and *** *p* < 0.001.

The transcriptional level of *hMSH2* in EGCG-treated COLO205 cells for 24 h was significantly up-regulated at 10, 20, and 40 μg/mL (*p <* 0.001). After 48 h treatment, no significant difference was found in the other concentrations except the 10 μg/mL group (*p <* 0.001). After 96 h of treatment, the 40 μg/mL group was significantly down-regulated (*p* < 0.001, Figure 7).

### 2.5. Discussion

EGCG exhibits antioxidant as well as pro-oxidant properties. Evidences in the literature suggest that the prooxidant activity of EGCG may account for its anti-proliferative and cancer therapeutic effects [26]. We observe that EGCG treatment can significantly increase the frequency of CIN in COLO205 cells (Table 2, Figure 3). The reason may be that EGCG leads to COLO205 cell cycle arrest. It has been reported that EGCG in multiple types of cancer cells induced mitotic arrest [27,28,29,30]. Prolonged mitotic arrest leads to abnormal spindle structure, chromosomal aberrations, sister chromatid disruption defects, centrosome abnormalities, and cytokinesis defects that ultimately result in MN, NPB, and multinuclear cells that contain excessive CIN, which can delay their entry into the next mitosis and extend the entire cycle of division, further triggering large-scale CIN. The results of this experiment showed that there was a significant negative correlation between the incidence of CIN and NDI (r = −0.861, *p <* 0.001).

Another mechanism by which EGCG induces CIN in COLO205 cells may be by disrupting its telomerase activity. A study had shown that with 70 μM of EGCG treatment of small-cell lung carcinoma (SCLC) for 24 h resulted in 50–60% reduced telomerase activity [31]. Decreased telomerase activity leads to shortened telomere length, inducing breakage-fusion-bridge circulation. The formation of NPB may be because the telomere at the end of the chromosome is too short (telomere crisis), or due to fusion to form a dicentric chromosome. In our study, NPB rates increased in a time-/dose-dependent manner after EGCG treatment in COLO205 cells (Table 2), suggesting that EGCG has the effect of inhibiting telomerase activity.

Recent studies have shown that excessive CIN in cancer cells can also lead to spontaneous death processes such as apoptosis [32]. There was a significant positive correlation between CIN and apoptosis rate in COLO205 cells (r = 0.949, *p <* 0.001), suggesting that one of the mechanisms by which EGCG inhibits tumor growth may be to increase the CIN rate of cancer cells beyond that suitable for their growth critical value, thus triggering apoptosis. Presumably, auto-oxidation of EGCG-generated ROS may have broken the redox balance of COLO205 cells.

On the other hand, EGCG can significantly reduce the MN, NBUD, and CIN rates in NCM460 cells (Table 1, Figure 2). Since excessive CIN can initiate cell carcinogenesis, the above data indicate that EGCG may maintain genomic stability by decreasing CIN in normal cells. This may be a cancer chemopreventive mechanism of EGCG. EGCG may show antioxidant activity in NCM460 cells. Epidemiological study found that repeated consumption of tea and encapsulated tea extracts for one to four weeks decreases the biomarkers of oxidative status, such as oxidative DNA damage, lipid peroxidation, and free radical solution [1]. EGCG has an inhibitory effect on DNA methyltransferase [33] and its activity results in up-regulation of gene expression to maintain genome stability, such as *hMLH1* and *hMSH2* (Figure 6). At the same time, EGCG treatment did not alter the genome methylation pattern of the cell lines tested (data not shown). EGCG may inhibit apoptosis and promote the proliferation of NCM460 cells by decreasing the CIN of cells and regulating the expression of related genes.

In conclusion, EGCG-induced chromosome instability may be the mechanism of selectively induced apoptosis in COLO205 cells. It seemed that the discrepancy of chromosome stability would be due to the differential inducibility of ROS, especially H_2_O_2_, by EGCG between cancer cells and normal cells. That is, increased perturbation of redox and ROS homeostasis by EGCG-induced ROS in cancer cells would be the base for the selectivity of EGCG-induced chromosome instability and apoptosis in cancer cells.

## 3. Materials and Methods 

### 3.1. Cell line and Cell Culture

NCM460, a cell line derived from human normal colon mucosal epithelial cells, was obtained from INCELL (San Antonio, TX, USA). Colo205, a colon adenocarcinoma cell line, was obtained from the Cell Bank of the Kunming Institute of Zoology (Chinese Academy of Sciences, Kunming, China). NCM460 and COLO205 cells were maintained as monolayers in 75-cm^2^ flasks in RPMI 1640 medium (Gibco, Gran Island, NY, USA) supplemented with 10% (*v*/*v*) new born calf serum (Gibco, Gran Island, NY, USA), 1% (*v*/*v*) penicillin (5000 IU/mL)/streptomycin (5 mg/mL) solution (Gibco, Gran Island, NY, USA), and 1% (*v*/*v*) L-glutamine (2 mmol/L) (Sigma, St. Louis, MO, USA), and kept at 37 °C in a humidified atmosphere containing 5% CO_2_.

### 3.2. Trypan Blue Exclusion Assay

COLO205 cells were seeded into 24-well plates (Corning, NY, USA) at a density of 1 × 10^5^ cells/mL and exposed to different concentrations of EGCG (0, 5, 10, 40, 80 μg/mL). After 24 h incubation, cell suspensions were stained with trypan blue (Boster, Wuhan, China) for 2 min and then counted using a hemocytometer. This procedure repeated three times in duplicate for each EGCG concentration.

### 3.3. CBMN Assay

CBMN assay was performed as previously described [34]. In brief, NCM460 and COLO205 cells were seeded into 24-well plates at a density of 1 × 10^5^ cells/mL and cultured in RPMI1640 medium containing 0, 5, 10, or 40 μg/mL EGCG for 24, 48, 72, or 96 h. At each sampling time, duplicate 400 µL subcultures were established in a new 24-well plate from the main cultures, at a concentration of 1 × 10^5^ cells/mL. Following a 2-h incubation period, cytochalasin B (4.5 µg/mL; Sigma) was added to block cytokinesis and rinsed with PBS after a further 24 h. Cells were harvested by trypsinization and centrifuged onto glass slides using a cyto-centrifuge for 5 min at 800 rpm (100 g). After drying briefly in air, slides were fixed in Carnoy’s Fluid and stained with 10% Giemsa. The slides were washed twice in ddH_2_O, and then allowed to air dry and a cover slip was added. Stained slides were encoded to ensure a blind microscopic analysis, and such a code was removed until the whole microscopic analysis was finished. All biomarkers of CBMN assay were scored under 1000× magnification with optical microscope (Olympus, Tokyo, Japan) using previously described criteria. A thousand binucleated cells (BNCs) were scored per group to determine the frequency of MN, nucleoplasmic bridge (NPB), and nuclear bud (NBUD).

### 3.4. Cell Proliferation and Apoptosis Analysis

After treatment, the cell suspensions were centrifuged to slides, fixed, and stained as mentioned above. The nuclear division index (NDI) is a more ideal method for detecting cell proliferation status. Cell division status was assessed by measuring the ratio of monocytes (non-dividing), binucleated cells (dividing only once), and multinucleated cells (multiple divisions) after Cyt-B treatment. The analysis of apoptosis was performed on the same slides. As defined previously, cells with chromatin condensation and intact cytoplasmic and nuclear boundaries as well as cells exhibiting nuclear fragmentation into smaller bodies within an intact cytoplasmic membrane were classified as apoptotic. In each sampling, a total of at least of 500 cells were scored.

### 3.5. Real-Time Quantitative PCR

After EGCG treatment, total RNA was prepared with high pure RNA isolation kit (Roche, Basel, Switzerland), which was utilized to synthesize cDNA with transcriptor first strand cDNA synthesis Kit (Roche, Basel, Switzerland) according to the manufacturer’s protocol. RT-qPCR was performed in triplicates using the Kapa SYBR fast qPCR kit (KAPA Biosystems, Woburn, MA, USA) and Applied Biosystems StepOne Plus RT-qPCR system (ABI, Foster, CA, USA). The expression of core genes in mismatch repair (*hMLH1* and *hMSH2*) was analyzed. The primer sequence was: 5′-GTGCTGGCAATCAAGGGACCC-3′ and 5′-CACGGTTGAGGCATTGGGTAG-3′ for *hMLH1*, 5′-ATCCAAGGAGAATGATTGGTATTTG-3′ and 5′-CAAAGAGAATGTCTTCAAACTGAGAGA-3′ for *hMSH2*, 5′-AACGTGTCAGTGGTGGACCTG-3′ and 5′-AGTGGGTGTCGCTGTTGAAGT-3′ for GAPDH. The samples were heated at 95 °C for 3 min followed by 40 cycles at 95 °C for 3 s and 60 °C for 20 s. Expression of these genes was normalized to the expression of GAPDH in each sample and fold change was calculated using the 2^−ΔΔCt^ method.

ΔΔCt = ΔCt_test group_ − ΔCt_control group_.

ΔCt = Ct_target gene_ − Ct_GAPDH_.

### 3.6. Statistical Analysis

All statistical analyses were performed using SPSS 17.0 for windows (SPSS, Chicago, IL, USA). The differences of observed values among the control and EGCG treated groups were analyzed using One-way analysis of variance (ANOVA). Two-way Jonckheere–Terpstra test was used to examine possible dose–response relationships between time points and EGCG treatment. We considered differences with *p*-value (two-tailed) lower than 0.05 to be significant. All the figures were graphed by GraphPad PRISM 5.0 (GraphPad, San Diego, CA, USA). All data are presented as means ± standard error of the mean of at least three independent experimental units.

## 4. Conclusions

EGCG significantly reduced chromosome instability and apoptosis rate of NCM460 cells at all concentrations (5–40 μg/mL) and treatment times. EGCG at 5 μg/mL promoted cell division. EGCG could significantly induce chromosome instability in COLO205 cells and apoptosis, inhibition of cell division, and the performance of time and dose effects. These results suggest that EGCG exhibits different genetic and cytological effects in normal and cancer cells.

## Figures and Tables

**Table 1:** The effects of EGCG on frequencies of CIN (MN, NPB, NBUD) in binucleated NCM460 cells.

CIN Index	Exposure Time	EGCG Dose (μg/mL)				
		0	5	10	20	40
(a) MN	24 h	65.40 ± 1.51	55.60 ± 1.50 ***	48.05 ± 2.34 ***	39.54 ± 0.91 ***	34.48 ± 1.51 ***
	48 h	61.57 ± 0.53	46.98 ± 1.67 ***	38.55 ± 1.62 ***	29.31 ± 1.81 ***	24.86 ± 1.81 ***
	72 h	53.51 ± 3.16	40.13 ± 1.76 ***	33.50 ± 2.55 ***	27.35 ± 1.65 ***	25.30 ± 1.54 ***
	96 h	73.97 ± 2.38	52.56 ± 2.13 ***	41.03 ± 1.70 ***	27.59 ± 2.37 ***	28.25 ± 3.58 ***
(b) NPB	24 h	9.30 ± 0.58	8.27 ± 0.49	9.28 ± 1.54	9.23 ± 0.65	8.95 ± 1.02
	48 h	9.02 ± 0.45	8.93 ± 0.99	8.58 ± 0.64	7.90 ± 0.95	7.62 ± 0.55 *
	72 h	10.50 ± 1.21	9.79 ± 1.02	9.29 ± 0.60	7.91 ± 1.06 *	7.99 ± 0.99 *
	96 h	9.62 ± 1.18	8.98 ± 0.99	8.60 ± 1.10	7.88 ± 0.88	7.64 ± 0.60 *
(c) NBUD	24 h	28.88 ± 0.95	26.15 ± 1.09 *	24.19 ± 1.64 ***	23.06 ± 0.42 ***	20.90 ± 1.02 ***
	48 h	20.32 ± 1.91	19.52 ± 2.04	17.78 ± 1.84	15.16 ± 1.62 **	13.26 ± 1.52 ***
	72 h	20.35 ± 1.33	16.97 ± 2.10 *	13.27 ± 1.52 **	10.87 ± 0.89 ***	10.65 ± 2.52 ***
	96 h	24.55 ± 2.89	20.29 ± 2.08 **	17.53 ± 2.93 **	11.49 ± 1.36 **	10.63 ± 2.10 **

Note: Data represented the mean ± S.E. per 1000 BNC from three independent experiments. Significant differences between EGCG-treated groups and controls at each treatment interval are indicated by * *p* < 0.05, ** *p* < 0.01 and *** *p* < 0.001.

**Table 2:** The effects of EGCG on frequencies of CIN (MN, NPB, NBUD) in binucleated COLO205 cells.

CIN Index	Exposure Time	EGCG Dose (μg/mL)				
		0	5	10	20	40
(a) MN	24 h	25.63 ± 1.16	30.24 ± 1.54 **	34.75 ± 0.88 ***	38.82 ± 1.11 ***	44.66 ± 1.22 ***
	48 h	24.22 ± 1.13	32.69 ± 0.93 ***	40.78 ± 1.93 ***	46.68 ± 1.22 ***	55.34 ± 1.69 ***
	72 h	21.58 ± 1.50	29.70 ± 1.33 ***	37.47 ± 0.91 ***	47.69 ± 1.09 ***	61.17 ± 2.48 ***
	96 h	28.57 ± 1.48	35.64 ± 1.11 **	43.81 ± 1.60 ***	62.54 ± 2.12 ***	NA
(b) NPB	24 h	15.45 ± 1.60	15.62 ± 1.53	15.56 ± 1.47	16.25 ± 0.61	18.19 ± 1.11 *
	48 h	19.31 ± 1.95	23.45 ± 2.02 *	26.86 ± 1.96 ***	30.79 ± 1.15 ***	37.45 ± 1.71 ***
	72 h	13.28 ± 1.54	23.41 ± 2.27 ***	32.55 ± 2.04 ***	41.43 ± 1.61 ***	51.53 ± 2.45 ***
	96 h	21.26 ± 1.57	27.39 ± 1.33 **	34.92 ± 2.09 ***	58.57 ± 1.17 ***	NA
(c) NBUD	24 h	5.92 ± 1.03	5.98 ± 1.73	5.96 ± 1.01	7.63 ± 0.60	9.92 ± 0.93 **
	48 h	9.16 ± 0.46	9.91 ± 0.99	11.60 ± 0.57 *	11.25 ± 1.46 *	12.26 ± 0.52 **
	72 h	8.63 ± 1.51	8.91 ± 0.99	9.20 ± 2.47	12.17 ± 1.60 *	11.64 ± 0.61 *
	96 h	14.95 ± 1.03	17.15 ± 2.00	20.09 ± 0.52 *	24.82 ± 2.01 ***	NA

Note: Data represented the mean ± S.E. per 1000 BNC from three independent experiments. Significant differences between EGCG-treated groups and controls at each treatment interval are indicated by * *p* < 0.05, ** *p* < 0.01 and *** *p* < 0.001. NA: not analyzable, cells cultured in 40 μg/mL EGCG for 96 h had a low NDI (1.089), resulting in too few countable BN cells to analyze.
